# Tissue-Level Integration Overrides Gradations of Differentiating Cell Identity in Beetle Extraembryonic Tissue

**DOI:** 10.3390/cells13141211

**Published:** 2024-07-18

**Authors:** Katie E. Mann, Kristen A. Panfilio

**Affiliations:** 1 School of Life Sciences, University of Warwick, Coventry CV4 7AL, UK; 2 Department of Molecular Genetics, Institute of Biology, University of Hohenheim, Garbenstr. 30, 70599 Stuttgart, Germany

**Keywords:** extraembryonic tissues, tissue differentiation, insects, polyploidy, RNAi, quantitative live imaging

## Abstract

During animal embryogenesis, one of the earliest specification events distinguishes extraembryonic (EE) from embryonic tissue fates: the serosa in the case of the insects. While it is well established that the homeodomain transcription factor Zen1 is the critical determinant of the serosa, the subsequent realization of this tissue’s identity has not been investigated. Here, we examine serosal differentiation in the beetle *Tribolium castaneum* based on the quantification of morphological and morphogenetic features, comparing embryos from a *Tc-zen1* RNAi dilution series, where complete knockdown results in amnion-only EE tissue identity. We assess features including cell density, tissue boundary morphology, and nuclear size as dynamic readouts for progressive tissue maturation. While some features exhibit an all-or-nothing outcome, other key features show dose-dependent phenotypic responses with trait-specific thresholds. Collectively, these findings provide nuance beyond the known status of Tc-Zen1 as a selector gene for serosal tissue patterning. Overall, our approach illustrates how the analysis of tissue maturation dynamics from live imaging extends but also challenges interpretations based on gene expression data, refining our understanding of tissue identity and when it is achieved.

## 1. Introduction

Early patterning decisions form critical checkpoints in development. Changes in patterning can drastically alter tissue identity, structure, and function, as well as the chances of survival [1,2,3,4]. Furthermore, the progression from specification to tissue differentiation offers opportunities for the integration of multiple genetic inputs that can refine or alter upstream specification [5,6,7]. While certain developmental events require threshold amounts of key patterning genes, particularly for robust patterning along a morphogen gradient (e.g., [8,9,10,11,12]), other aspects can proceed in a modular, independent fashion [13,14]. Thus, investigating the phenotypic effects of patterning gene manipulation can distinguish the mechanisms required for the full spectrum of tissue differentiation.

The differentiation of the extraembryonic (EE) tissues represents a key system for investigating patterning decisions during animal embryogenesis. These tissues are among the earliest to differentiate. Their rapid maturation is implicated in the evolutionary success of amniote vertebrates and insects, due to their essential roles in protecting and provisioning the embryo [15,16,17]. Specific properties of the fully differentiated tissues are tightly linked to their protective functions. For example, mouse syncytiotrophoblasts and the two insect EE tissues—amnion and serosa—switch from mitosis to the endocycle and become polyploid, with tissue-specific levels of ploidy [18,19,20,21,22,23]. Polyploidy in EE tissues is hypothesized to provide mechanical and physiological protection, as the large nuclei support tissue integrity and protection against infection, including via increased expression of signaling and effector molecules for innate immunity [23,24,25,26]. Additionally, the insect serosal tissue, as the outermost cellular layer in the egg, secretes a thick chitin-based cuticle that enhances eggshell strength and desiccation resistance [27,28,29,30,31,32,33].

In insects, the homeobox gene *zen* (or *zerknüllt*), the diverged orthologue of *Hox3*, is well established as a marker gene for EE tissue [3,34,35,36], and there is clear evidence that it is the single critical EE determinant—a “selector gene”—in holometabolous species [1,37]. It specifies the amnioserosa as the single EE tissue in cyclorrhaphan flies including *Drosophila melanogaster* [38,39]. In fly species with a distinct serosa and a minimal amnion, Zen orthologues determine the early EE tissue region as serosal; subsequently, additional genetic inputs specify the amnion at the EE periphery and delimit the embryonic–extraembryonic tissue border [37,40,41,42].

**Figure 1:**
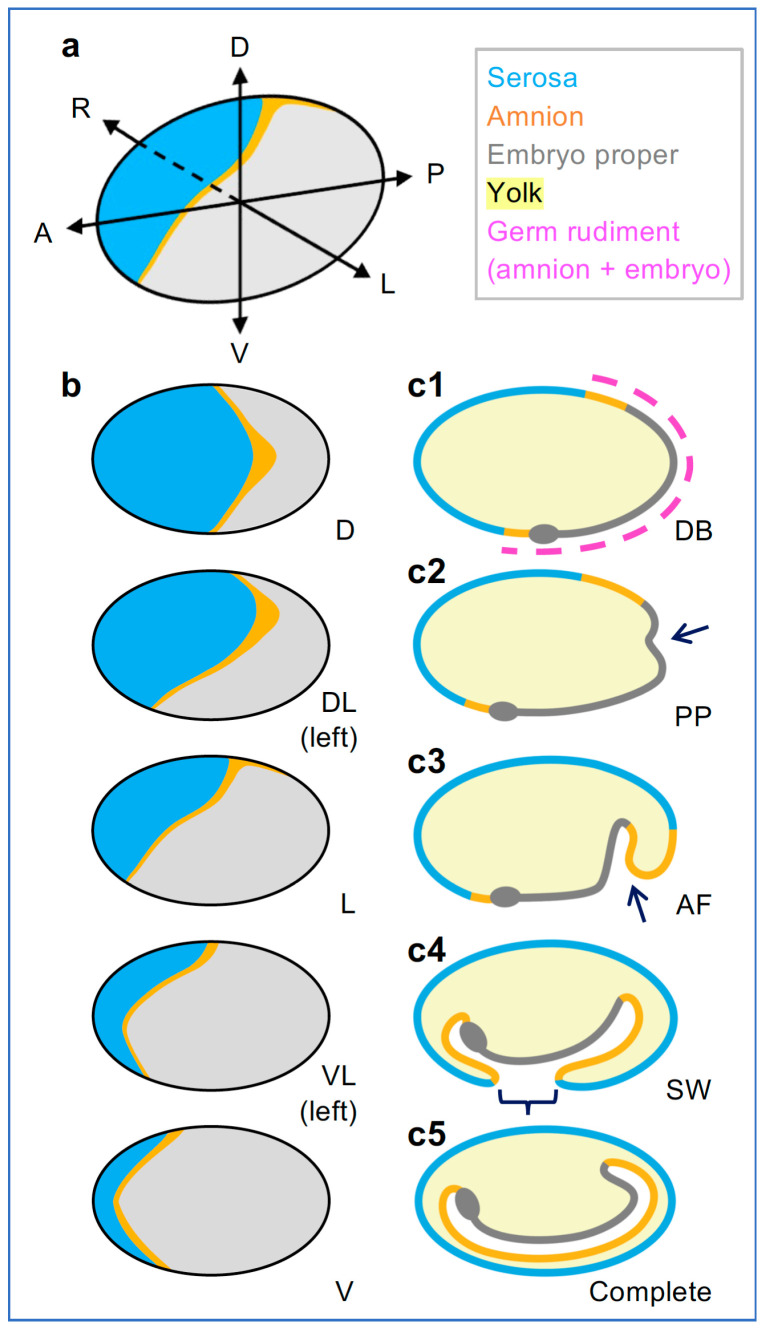
Early wild-type *Tribolium* fate map and extraembryonic (EE) morphogenesis. (**a**) Axial orientation and (**b**) surface views at the differentiated blastoderm stage. The angle of view affects the visible area of the anterior-dorsal EE domain. The presumptive amniotic region is based on gene expression and lineage tracing [1,49,50]. (**c**) Ensuing morphogenesis leads to the embryo being fully enclosed in an outer serosal and an inner amniotic layer (mid-sagittal views): arrows indicate initial apical constriction and deeper invagination; the curly bracket spans the open “window” region; gray ovals indicate the anterior of the embryo proper. Abbreviations: for egg axes: A-P, anterior–posterior; D-V, dorsal–ventral; L-R, left–right; for angles of view: D, dorsal; DL, dorsal–lateral; L, lateral; VL, ventral–lateral; V, ventral; for landmark stages: DB, differentiated blastoderm; PP, primitive pit; AF, amniotic fold; SW, serosal window. Morphogenesis schematics modified from [17].

**Figure 2:**
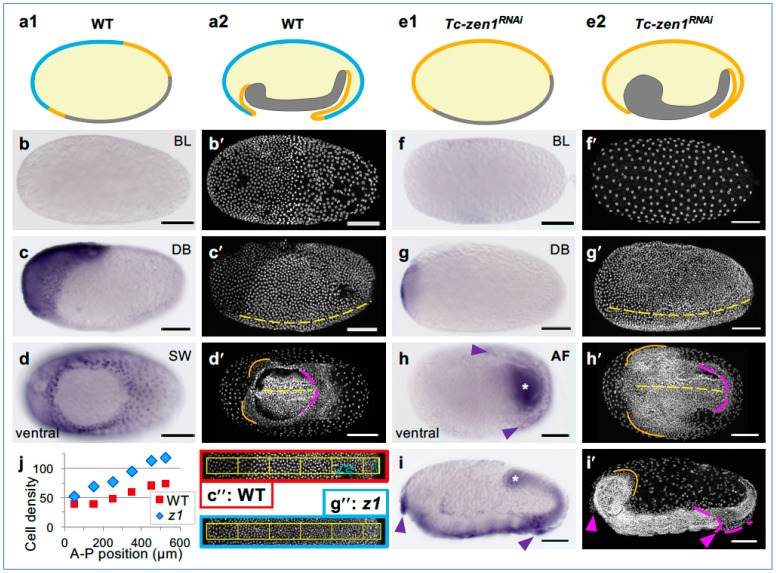
*Tc-hnt* expression reveals subtle tissue regionalization after *Tc-zen1* RNAi. Fate map, morphology, and expression of *Tc-hnt* in wild-type (WT) (**a**–**d**) and *Tc-zen1* RNAi (“*z1*”) (**e**–**i**) embryos. (**a1**,**a2**,**e1**,**e2**) Mid-sagittal schematics of the serosal (blue), amniotic (orange), and embryonic (gray) domains at the differentiated blastoderm and extending germband stages. (**b**–**d**,**f**–**i**) *Tc-hnt* in situ hybridization, with DAPI counterstain (letter-prime panels): blastoderm formation (**b**,**f**), differentiated blastoderm (**c**,**g**), and EE tissue fold formation ((**d**): closing serosal window, (**h**,**i**): persistent ventral-posterior amniotic fold, “AF”). Views are ventral–lateral (**c**,**g**), ventral (**d**,**h**), lateral (**i**), or indeterminate (**b**,**f**). Morphological annotations: yellow: ventral midline; magenta: posterior rim of the EE tissue enclosing the embryo; orange: head lobe rim; arrowheads: *Tc-hnt*-expressing folds of EE tissue after knockdown; asterisks: posteriormost extent of the embryo. Scale bars are 100 µm. (**j**,**c″**,**g″**) Knockdown embryos exhibit higher cell density throughout the differentiating blastoderm and yet retain lower density in the ATR, which coincides with residual *Tc-hnt* expression (measured in 100 × 50 µm sectors along the A-P axis, as depicted in (**c″**,**g″**) for the exemplar embryos shown in (**c**,**c′**) and (**g**,**g′**), respectively: double-prime images indicate measured sectors; density was scaled for sector size and to exclude minor damage: cyan regions).

In the red flour beetle, *Tribolium castaneum*, the orthologue *Tc-zen1* is strictly required for serosal identity in an oblique anterior–dorsal region of the blastoderm (Figure 1a,b; [1,5,43]). Subsequent morphogenesis leads to the mature EE tissue configuration: the outer serosa fully envelops the embryo, amnion, and yolk, while the inner amnion delimits a fluid-filled cavity ventral to the embryo (Figure 1c; [44,45,46]). This configuration represents the standard EE tissue complement in winged insects [15,47,48]. After *Tc-zen1* RNAi, loss of serosal identity leads to the respecification of the blastoderm, with an anterior–dorsal expansion of amniotic and embryonic head tissue identities (Figure 2(a1,e1), below; [1]).

However, the depth of characterization of EE development in *Tribolium* has led to intriguing observations that belie the apparent all-or-nothing character of serosal specification by Tc-Zen1. First, Tc-Zen1 acts upstream of or parallel to factors with dynamic expression waves that originate at the anterior pole and then pass posteriorly, exiting the presumptive serosal region and subsequently defining the adjacent presumptive amniotic tissue (e.g., *Tc-Dorsocross, Tc-iroquois*; [13,51]). Second, unlike cyclorrhaphan *bicoid* and lepidopteran *Shx* as exceptional Class 3 Hox genes with precise maternal localization [8,52,53,54], *Tc-zen1* is zygotically expressed, downstream of a battery of maternal patterning factors for anterior and terminal system identities [2,5,55]. Thus, we wondered how modulating levels of *Tc-zen1* would impact serosal tissue specification against this backdrop of dynamic anterior gene expression.

Here, we assess hallmark serosal features throughout differentiation. Our marker gene profiling highlights an ostensible contradiction between early subregionalization and final tissue fate. To go beyond this, we generated a phenotypic series for *Tc-zen1* knockdown and analyzed EE development by live cell imaging. Tracking active dynamics and quantifying transient tissue features comprise a powerful approach (e.g., [56]), and here we use this strategy to reveal subtle aspects of differentiation. We speculated that either the character of the presumptive serosal region shows progressive changes towards an amniotic identity with increasing dsRNA concentrations or that there is a distinct concentration threshold for serosal identity. In fact, we observe a series of thresholds for distinct aspects of differentiation, which can result in a transient hybrid cell character, before the modular onset of EE identity progresses to a coherent, tissue-scale outcome.

## 2. Methods

### 2.1. *Tribolium castaneum* Stocks

*Tribolium castaneum* (Herbst) beetles were maintained under standard conditions at 30 °C and 40–60% RH [57]. The strains used were San Bernardino (SB) wild type [57], nuclear GFP (nGFP) [58], and enhancer trap lines for the serosa (G12424, KT650), amnion (HC079), and cardioblasts/embryonic segmental domains (G04609) [46,59,60]. SB was used for in situ hybridization; the nGFP and enhancer trap lines were used for fluorescent imaging.

### 2.2. RNAi and In Situ Hybridization

Linear PCR amplicons served as templates for double-stranded RNA (dsRNA) (Ambion T7 Megascript kit, ThermoFisher Scientific, Darmstadt, Germany) and in situ hybridization probes (Roche DIG RNA Labeling Mix and T7 RNA Polymerase kits, Sigma/Merck, Darmstadt, Germany), as described [13,46]. Sequences were based on the official gene set OGS 3 [61]. Primer sequences are listed in Table S1, including adapters for adding T7 promotor sites (as in [46]).

For RNAi, dsRNA for *Tc-zen1* (TC000921) used a 203 bp fragment for the embryonic RNAi (eRNAi) dsRNA dilution series (as in [5,62]) and a 688 bp fragment for parental RNAi (pRNAi) in marker (trans)gene assays (as in [13,59]). Previously, we showed that at 1 µg/µL, either dsRNA fragment achieves knockdown to 10–11% of wild-type *Tc-zen1* mRNA levels, as assayed by RT-qPCR [5]. The experimental controls were uninjected adults or eggs (negative control) and RNAi for an unrelated gene that produced a distinct knockdown phenotype (positive control: conserved cell cycle regulator *Tc-double parked* (*Tc-dup*), TC003416, 373 bp amplicon, also known as *Cdt1* [63]; phenotype of severe blastoderm mitosis impairment). For eRNAi, the dsRNA concentrations were 100 ng/µL, 200 ng/µL, and 750 ng/µL for *Tc-zen1* and 250 ng/µL and 1 µg/µL for *Tc-dup*, with consistent and minimal injection wound leakage, ensuring reasonable accuracy for dsRNA delivery. For pRNAi, *Tc-zen1* dsRNA was injected at 1 µg/µL. At least two biological replicates were performed for each RNAi treatment. For eRNAi, pre-blastoderm eggs (2–3 h after egg lay, hAEL) were dechorionated in bleach (“DanKlorix Hygienereiniger”, Colgate-Palmolive: 4–5% sodium carbonate and 1–4% sodium hypochlorite), mounted in halocarbon oil 700 (Sigma/Merck, Darmstadt, Germany), and injected anteriorly, as described [13]. For pRNAi, dsRNA was injected into the abdomen of adult females [32,59]. 

For whole-mount in situ hybridization, *Tc-hindsight* (*Tc-hnt*, TC009560) was detected with a 627 bp probe [13]. Sense strand probe detection served as a negative control, while the previously characterized amniotic marker *Tc-pannier* was used as a positive control (*Tc-pnr*, TC010407, [1,49], 757 bp probe). Colorimetric detection was as described [13,46]. Control eggs from uninjected mothers were stained in parallel with the *Tc-zen1* RNAi eggs, including matched duration of the alkaline phosphatase reaction for NBT-BCIP detection. All probes were used in at least three independent experiments. Eggs were imaged in Vectashield mountant with DAPI (Vector Laboratories, Newark, CA, USA), as described [5]. Focal stacking for in situ images was either performed manually in Photoshop (Adobe, San Jose, CA, USA) or with Helicon Focus (v.7.5.8, Helicon Soft, Kharkiv, Ukraine).

### 2.3. Live Imaging Acquisition and Analysis

Time-lapse movies were acquired with a Zeiss AxioImager.Z2 with an Apotome.2 structured illumination epifluorescent microscope (Carl Zeiss, Jena, Germany), as described [32], at 26–28 °C at intervals of 15 or 30 min. Data handling was performed in Image J (NIH, version 1.53k), including cell tracking with the MTrackJ plugin [64]. Unscorable eggs (e.g., unfertilized, non-specific defects, or xyz position drift that precluded accurate spatiotemporal quantification across developmental stages) were excluded from subsequent analyses. Maximum-intensity z-projected images were used for all analyses. Eggs were categorized by the angle of view to ensure consistent comparisons. Sample sizes and numerical results are specified in the figure legends and Supplementary File S1 (see tab “eRNAi overview stats” for all experiments).

#### 2.3.1. Spatial and Temporal Analyses

Early embryos were manually labeled at the boundary between the non-dividing EE tissue with low cell density and the condensing germ rudiment tissue with high cell density (Figure 4, below). Traces of the boundaries were then overlayed to illustrate the curvature and variation in border shape per treatment. For sufficient sampling, embryos that varied between strictly dorsal and dorsal–lateral angles of view were included, which is reflected in minor skewing in the traces. EE tissue area was normalized to egg area (μm^2^) from the z-projected images. Aspect ratios were based on the maximum axial extent of the EE tissue relative to the total egg length (anterior–posterior, A-P) and width (dorsal–ventral, D-V).

The timing of blastoderm differentiation was when the germ rudiment-specific mitotic division occurred. If the differentiation occurred over several time frames of the movie, the median frame was taken. The formation of the posterior amniotic fold was determined as the earliest time frame when the invaginating primitive pit had deepened sufficiently to form a ventrally oriented tissue crest that could be consistently discerned from multiple angles of view. Movie time frames were converted into age (hAEL) to directly compare treatments, based on the minimum age from a 1-h egg collection.

#### 2.3.2. Nuclear Area and Cell Density

To measure nuclear size (apical area on z-projected images), contrast settings were adjusted for the consistency of GFP appearance across embryos, and nuclei were manually outlined with the polygon tool in ImageJ, excluding nuclei at the periphery to avoid skewing due to egg curvature. In order to establish baseline differences between EE and germ rudiment tissue, 3 nuclei were measured for both tissues in 6–16 embryos per treatment condition. When measuring EE tissue alone at selected stages, 10 nuclei were measured per embryo from the same dataset. To ensure balanced sampling across the EE tissue, at the blastoderm differentiation stage, nuclei were randomly chosen within each of four equally sized subregions (illustrated in Figure S1). At the extended germband stage (20 hAEL), nuclei covering the yolk were selected from throughout the tissue (as illustrated in Figure S3d). Lastly, for the purposes of tracking changes in EE nuclear size over time, 5 nuclei were tracked for 3 embryos per treatment condition 

Cell (nuclear) density in the mature EE tissue (20 hAEL) was determined with the multi-point tool in Image J, within three square regions (80 µm × 80 µm; illustrated in Figure S2). Counts included nuclei for which >50% of the nuclear area was within the bounding box. For statistical analysis, the mean count from the three regions per embryo was used.

#### 2.3.3. Head Lobe Area and Cell Density in the EE–Embryonic Tissue Border Region

Morphology was quantified for head size and EE rim cell packing—the transition in cell density from embryonic to EE tissue in the region of the head lobes. Head area (µm^2^) was measured at 18–20 hAEL (at the extended germband stage, morphologically matched by eye within this stage range/time) for one head lobe fully visible in lateral aspect. The head region was delimited as follows: the dorsal surface was taken as the maximum region within which cell density was sufficiently high that individual nuclei could not be distinguished. The posterior extent of the head was delimited by the inflection point in shape between the head lobe and the rest of the germband. The ventral extent was measured as follows: to account for minor differences in angle, the region of interest for the head area was measured either to the ventral edge of the egg or to the morphologically distinct ventral midline, whichever was the smaller region.

To systematically sample EE cell density radiating out from the condensed head tissue of the embryo (illustrated in Figure 7a′,b′, below), five rectangular regions of interest (85 × 16 µm) were equally spaced, radiating in step sizes of 25°. Rectangles were positioned adjacent and tangential to the dense embryonic tissue region, defined as the region within which nuclear density was too great to distinguish individual nuclei. Nuclei were counted as described above (including nuclei with ≥50% of the total nuclear area inside the region of interest).

### 2.4. Statistical Analysis

Shapiro–Wilk tests determined the normality of the data. For non-normally distributed data that required >2 comparisons, the Kruskal–Wallis test (non-parametric one-way ANOVA) was used to compare the distribution of the data, with Dunn’s post hoc analysis for pairwise comparisons of means using rank sums. Bonferroni correction was used to adjust the significance values for multiple comparisons. The Mann–Whitney U test was used for data from two independent samples. For normally distributed data that required >2 comparisons, the one-way ANOVA test for variation in the means, with post hoc Tukey HSD (honestly significant difference) testing for pairwise significance, was used. Independent sample *t*-tests were used for comparisons between two treatment groups only. Statistical tests were conducted in GraphPad Prism (version 9.5.0) and graphs were made in Microsoft Excel (version 16.0). In the figures, the significance levels are as follows: *: *p* < 0.05; **: *p* < 0.01; and ***: *p* < 0.001; ns, not significant. Box plots depict the mean (“X”), median (horizontal line), and Q1–Q3 interquartile range (box height), while bars and whiskers denote the full data range, with outliers plotted individually. All values for graphs and the statistical test details are provided in Supplementary File S1.

Due to the inverse relationship between the *p*-value and observed power, by definition non-significant results will have low observed power, virtually independent of the sample size [65,66]. We used G*Power (version 3.1.9.7, [67]) to assess the likelihood of Type II errors (i.e., errors when one fails to reject a null hypothesis that is actually false, [68,69]). These calculations are presented in Supplementary File S1 (tab “non-significance assays”), and we interpret our non-significant results below with due caution.

## 3. Results

### 3.1. Early Morphogenetic and Morphological Differentiation of the Serosa

Early embryogenesis has been well characterized in *Tribolium* [1,5,13,44,45,62,70]. Briefly, the formation of the uniform blastoderm (BL) as a continuous epithelium over the yolk culminates in the completion of cellularization and optimization of cell packing (Figure 2b,b′; [71,72]). Then, the 13th cell division results in the differentiated blastoderm (DB), distinguishing the germ rudiment, which remains mitotically active, from the serosa, which ceases mitosis and enters the endocycle (Figure 1(c1)). In parallel, early morphogenesis begins. This involves rapid condensation of the embryo and its invagination into the yolk, while serosal epiboly maintains tissue continuity over the yolk surface. The key morphogenetic features are: (1) the formation of the primitive pit (PP) via apical constriction at the posterior pole, initiating invagination; (2) the anteriorly advancing ventral amniotic fold (AF) that engulfs the embryo; and (3) the final closure of the serosal window (SW) opening, utilizing an EE supracellular actomyosin cable and including contributions from minor anterior and lateral EE folds (Figure 1(c2–c4)). Severing their physical connection, the serosa and amnion then form a double membrane surrounding the embryo proper, producing the mature EE tissue topology (Figure 1(c5) and Figure 2(a2)) by 12 hAEL (17% of embryogenesis, [46]).

**Figure 3:**
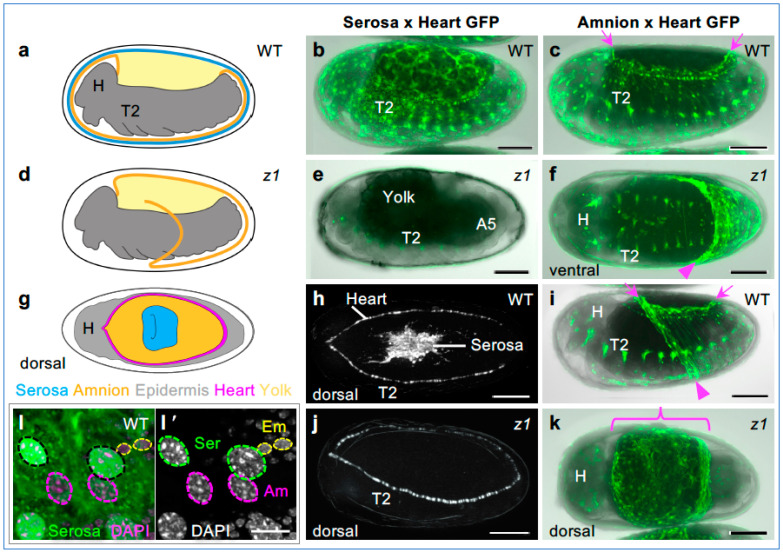
Strong *Tc-zen1* knockdown results in amnion identity in mature EE tissue. EE tissue structure and identity in WT (**a**–**c**,**g**–**i**,**l**) and after *Tc-zen1* RNAi (**d**–**f**,**j**,**k**): (**a**–**f**,**l**) retracting and retracted germband stages, (**g**–**k**) EE tissue withdrawal and dorsal closure stages. Views are lateral unless otherwise indicated. In the schematic drawings, the outermost dark gray layer is the eggshell (vitelline membrane). In the micrographs, GFP labels either EE tissue and a heart/segmental marker in heterozygous crosses, as indicated, in live (**b**–**k**) or fixed embryos (**l**). The GFP signal overlaid on brightfield shows embryonic tissue as a translucent gray and the yolk as an opaque black. During dorsal closure, the cardioblasts delimit the EE region, obviating the need for brightfield (see also Movies S1 and S2). Arrowheads indicate amnion folded edges: of the persistent *Tc-zen1* RNAi ventral pouch (**f**) or the transient WT ventral sac during mid-withdrawal (**i**). Before dorsal closure, the WT amnion does not cover the yolk ((**a**,**c**,**i**): arrows), while knockdown EE tissue fully spans the dorsal region and is amnion-GFP-positive ((**d**,**k**): curly bracket). Nuclear outlines are based on DAPI counterstain (**l′**); only serosal nuclei are GFP-positive (**l**). Abbreviations: Am, amnion; Em, embryo; H, head; Ser, serosa; T2, second thoracic segment. Scale bars are 100 µm (**b**–**k**) and 10 µm (**l**).

Morphologically, blastoderm differentiation rapidly results in a visual distinction between the serosa and the germ rudiment (Figure 2c,c′,c″). As morphogenesis proceeds, serosal cells flatten and become squamous, with low cell density and large, polyploid nuclei—defining features of the serosa (Figure 2d,d′,j; [44,45]). After modest further mitosis during germband extension, the amnion also enters the endocycle, although amniotic nuclei remain smaller, with characteristically lower ploidy than the serosa (Figure 3; [32,50,59,73]).

### 3.2. Strong *Tc-zen1* RNAi Leads to Loss of Serosal Tissue Identity

After *Tc-zen1* RNAi, the blastoderm anterior is respecified, and early morphogenesis results in partial enclosure of the embryo by folds of the amnion alone (Figure 2(e1,e2); [1,32]). Here, we reproduce this full-strength *Tc-zen1* RNAi knockdown phenotype (Figure 2f–i), as described previously from pupal [1,5], adult [13,32], and embryonic [62] injections.

Key morphological hallmarks of this strong phenotype are germ rudiment-specific mitotic divisions occurring throughout an expanded region of the blastoderm (Figure 2g,g′,g′′,j) and characteristic head lobe and EE tissue folding morphology. Specifically, the dorsal–lateral rim of the head lobes exhibits diffuse cell packing (Figure 2h,h′) and remains uncovered by EE tissue folds (Figure 2i,i′). In contrast, the EE tissue forms an extensive ventral fold, or pouch, over the thorax and abdomen, and there is often a medial fold, or flap, of EE tissue that overhangs the head at the anterior pole (Figure 2h,i).

We further confirm that the EE tissue in the strong *Tc-zen1* knockdown embryos is amnion. This was previously shown with the early amniotic marker genes *Tc-pannier* (*Tc-pnr*) [1] and *Tc-Dorsocross* (*Tc-Doc*) [13], which expand throughout the EE tissue domain in *Tc-zen1* RNAi embryos. However, these genes are pleiotropic and later acquire embryonic expression domains. To determine the identity of the mature EE tissue after *Tc-zen1* RNAi, we conducted a live imaging screen with transgenic lines with tissue-specific GFP (Figure 3). We visualized either the serosa or the amnion in heterozygous crosses with an embryonic enhancer trap line that provided an imaging internal control, labeling the heart (cardioblasts) and body segments as anatomical landmarks (Figure 3a–c). We examined embryos throughout the middle 50% of embryogenesis (24–60 hAEL), from the extended germband through EE withdrawal and dorsal closure stages (Figure 3a,d,g; Movie S1). With two independent serosa-GFP lines, after *Tc-zen1* RNAi, we observe a complete absence of GFP in EE tissue (100%, n = 170, Figure 3b,e,h,j; Movie S2). In contrast, amnion-GFP is expressed throughout the knockdown EE tissue, including the early EE ventral pouch and the entire dorsal region during EE withdrawal (n = 47, Figure 3c,f,i,k). In mature EE tissues, wild-type serosal nuclei are two-thirds larger than amniotic nuclei (apical area), a distinction that persists after knockdown (Figure 3b,c,k,l,l′; [32]). Lastly, we detect no serosal cuticle (mechanical reinforcement of the eggshell) after *Tc-zen1* RNAi in any of the transgenic backgrounds (as in [5,30,32]).

Thus, tissue-specific transgenes as well as nuclear size and the absence of cuticle confirm the stable identity of all EE cells as amniotic after strong *Tc-zen1* RNAi, supporting the mid-embryogenesis morphological hallmarks as reliable indicators for this phenotype.

### 3.3. *Tc-zen1* RNAi Reveals Early, Subtle Regionalization within the EE Tissue

Despite the clear amniotic identity of *Tc-zen1* RNAi EE tissue, based on multiple genetic and morphological features, the serosal marker gene *Tc-hnt* reveals regionalization within the knockdown tissue that challenges a binary serosa/amnion tissue identity. Wild-type expression of *Tc-hnt* arises in the serosa during blastoderm differentiation and persists as a faithful marker of the entire tissue throughout germband stages (Figure 2b–d, [13,51]). After strong knockdown of *Tc-zen1* and loss of serosal identity, *Tc-hnt* is largely absent but is still detected in two specific domains (Figure 2f–i). First, *Tc-hnt* has restricted expression in morphogenetically active folds of the EE tissue (Figure 2h,i: arrowheads), consistent with the regulation of ectopic supracellular actin cables by *Tc-Doc* [13].

More intriguingly, after *Tc-zen1* RNAi, early *Tc-hnt* expression still occurs in a small, transient domain at the anterior pole (Figure 2g). This domain may reflect *Tc-zen1*-independent patterning [4,13,55]. Yet, this genetic distinction correlates with morphology that is characteristic of the serosa: cells in this region have larger nuclei (Figure 2g,g**′**) and notably lower cell density than the rest of the blastoderm (Figure 2c′′,g′′,j: anteriormost region; [1]). Thus, we wondered how varying the strength of *Tc-zen1* RNAi would alter this distinctive anterior terminal region (ATR) and resulting tissue specification.

### 3.4. A Phenotypic Spectrum for EE Tissue Differentiation

To examine spatiotemporal dynamics with accuracy of stage-matched comparisons between wild-type and knockdown embryos, and to move beyond marker gene analyses restricted to candidate (trans)genes (Figure 2 and Figure 3), we next undertook a quantitative live imaging approach to examine the EE tissue in a dilution series for *Tc-zen1* dsRNA. Our primary focus was on the morphological and morphogenetic features that define tissue identity, and particularly how this is manifest in the ATR. We conducted these experiments in a transgenic line in which ubiquitously expressed, nuclear-localized GFP fully labels nuclear volume [58]. This allowed us to visualize all nuclei (all cells) of the blastoderm, irrespective of differentiation status or subsequent tissue identity, and to quantify key diagnostic features such as the timing of mitosis, cell packing, and nuclear size (Movie S3).

**Figure 4:**
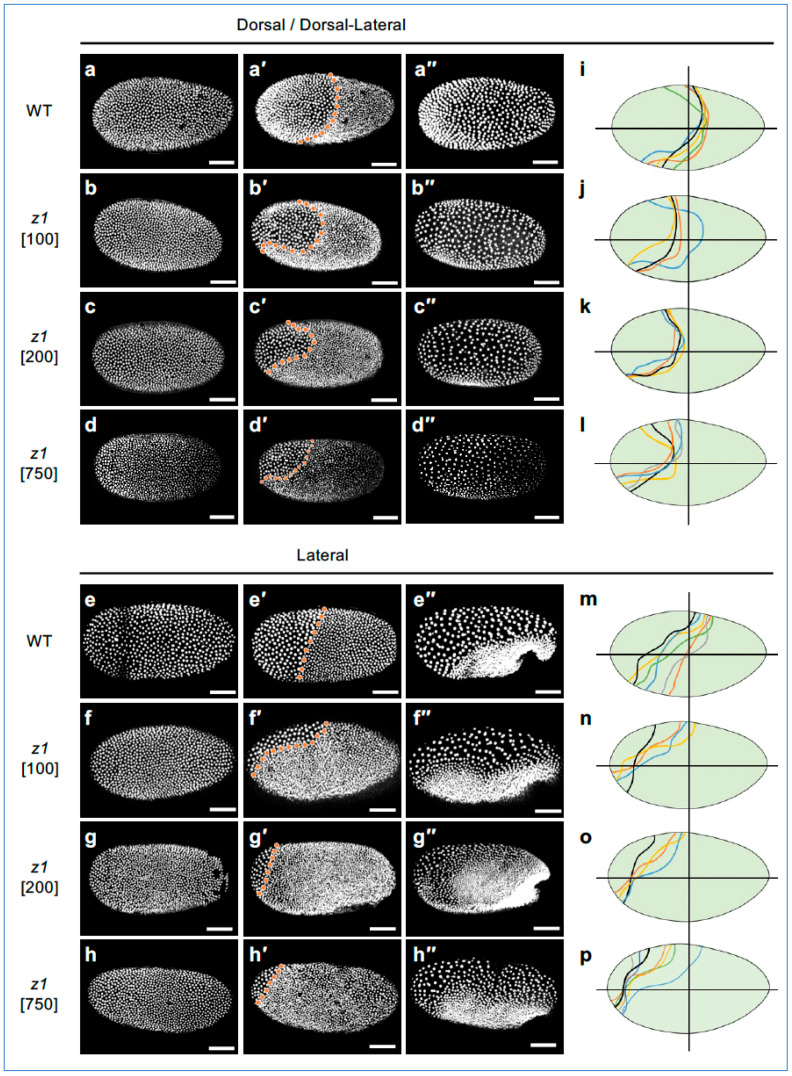
*Tc-zen1* dsRNA concentration-dependent reduction in the morphologically distinct EE domain. Qualitative analysis of EE tissue area at the uniform blastoderm (**a**–**h**), differentiated blastoderm (**a′**–**h′**), and the posterior fold stages (**a″**–**h″**). Orange dotted line marks the boundary between the EE and condensing embryonic tissues. EE tissue area, in both dorsal (**a**–**d**) and lateral (**e**–**h**) aspect, is greatest in WT (uninjected controls) and progressively reduces across the three dsRNA treatments (concentrations as indicated: 100, 200, or 750 ng/µL; see Figure 6 for quantification, and Movies S3–S7). (**i**–**p**) EE domain traces depicting boundary shape and curvature for all treatments at the differentiated blastoderm stage: each colored line represents one embryo in the dataset (see Methods for boundary determination). Horizontal lines indicate the A-P axis midline; vertical lines at 50% egg length provide a landmark for comparing EE tissue extent. Scale bars are 100 µm.

We first asked whether it is possible to achieve weaker knockdown phenotypes for *Tc-zen1*, and if so, what the consequences would be for blastoderm differentiation and early tissue morphogenesis. To explore this, we performed eRNAi by injecting young eggs with three distinct dsRNA concentrations—100, 200, or 750 ng/µL—balancing the need for statistically robust sample sizes with testing a range of dilutions. With our highest concentration, representing 75% of the dsRNA concentration previously used in both eRNAi [62] and pRNAi (e.g., Figure 2), we could reproduce the full-strength phenotype (Figure 4, cf. Figure 2 and further analyses below). Our lower concentrations represent five-fold and ten-fold dilutions compared to previous eRNAi full-strength *Tc-zen1* knockdown [62]. These concentrations did indeed produce a range of outcomes for EE tissue area, which is most evident during blastoderm differentiation (Figure 4a′–h′,i–p; Movies S4–S7).

**Figure 5:**
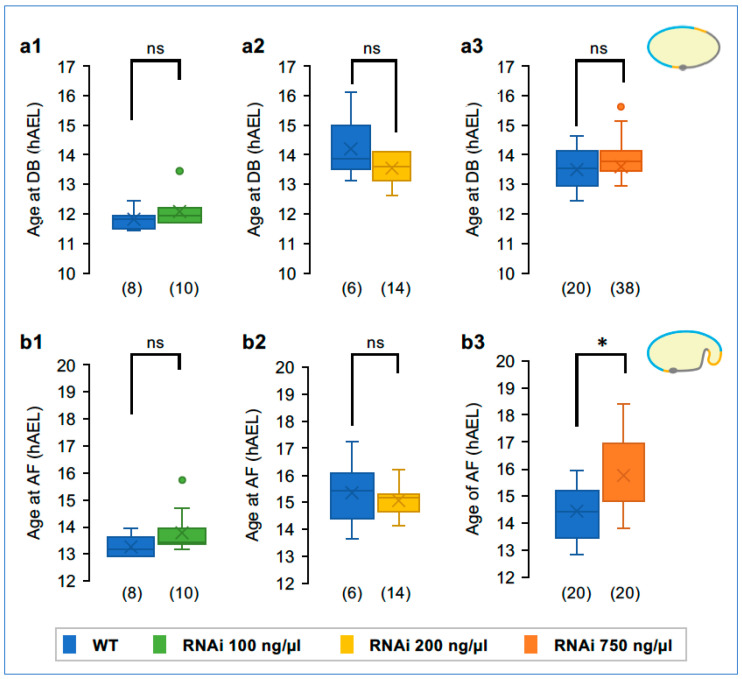
Timing of landmark early events. Timing of blastoderm differentiation ((**a1**–**a3**): DB) and amniotic folding ((**b1**–**b3**): AF). Only embryos in a strictly lateral view were analyzed for the latter. To mitigate interexperimental differences such as ambient temperature, knockdown embryos were directly compared to within-experiment WT controls. Significance was determined by Mann–Whitney U tests, revealing the onset of a delay during amniotic fold formation in the strongest knockdown treatment (*p* < 0.05). Sample sizes are indicated parenthetically; box plot features are defined in the Methods. See Supplementary File S1 for raw values and test statistics.

To ensure that our quantitative analyses of EE structure are directly comparable, we evaluated the timing of early developmental events. eRNAi for *Tc-zen1* does not significantly affect the timing of blastoderm differentiation with any of the three dsRNA concentrations (Figure 5(a1–a3)). As the wild-type controls were uninjected, this also provides evidence that the eRNAi treatment itself did not alter the rate of early development. In contrast, as the *Tc-zen1* RNAi phenotype manifests and develops, this led to a significant delay in EE tissue folding in the strongest knockdown treatment, by 17% development (Figure 5(b1–b3): an 80-min delay compared to the wild type, *p* = 0.012, Mann–Whitney U test; see Supplementary File S1 for all raw data values and test statistic details here and below).

### 3.5. Dose-Dependent and Non-Uniform Reduction in the EE Domain after *Tc-zen1* RNAi

To examine EE tissue size and shape, we first inspected embryos during early differentiation (Figure 4a–h″). Wild-type embryos (Figure 4a′,e′) have a large serosal domain with low cell density and large nuclei, whereas strong knockdown embryos retain only the small ATR of presumptive amniotic tissue (Figure 4d′,h′). Lower dsRNA concentrations produced EE areas of intermediate sizes (Figure 4b,b′′,c,c″,f,f″,g,g″). We noted natural, inter-embryo variability in EE tissue area, but did not observe any systematic left–right asymmetries or treatment-specific changes in boundary curvature (Figure 4i–p). Also, despite a lack of evident subregionalization within wild-type EE tissue (presumptive serosa), the EE tissue after strong knockdown (presumptive amnion, with 750 ng/µL) is reduced to the ATR.

**Figure 6:**
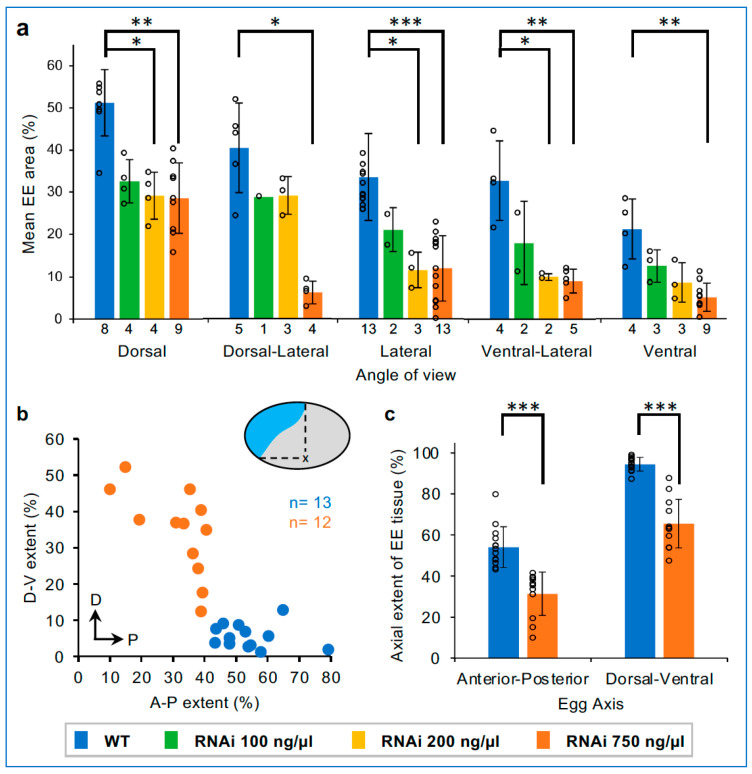
Non-uniform and threshold responses in knockdown EE area reduction. (**a**) Mean EE tissue area (% relative to egg size) at the differentiated blastoderm stage, for all angles of view for each RNAi condition, with sample sizes indicated at the base of the graph. Error bars represent ± one standard deviation; individual plot points are also shown. (**b**) An aspect ratio scatter plot for individual embryos in lateral aspect shows the relative reduction in EE tissue area along the A-P and D-V axes (sample sizes indicated in the plot). Compared to the WT serosa, after strong knockdown (dsRNA 750 ng/µL), the presumptive EE tissue does not extend as far ventrally or posteriorly. (**c**) Mean ± standard deviation summary statistics for the dataset in panel (**b**): same individual plot points shown. Significance levels are defined in the Methods; unlabeled pairwise comparisons were not significant. See Supplementary File S1 for raw values and test statistics ((**a**): Kruskal–Wallis with Dunn’s post hoc; (**c**): Mann–Whitney U).

Going further, we observe an overall graded decrease in EE tissue area with increasing concentrations of *Tc-zen1* dsRNA, with threshold sensitivities depending on the angle of view (Figure 6a). The wild-type serosa covers half the dorsal surface area and one-fifth of the ventral side (Figure 1 and Figure 6a). The strongest knockdown treatment (750 ng/µL) resulted in a significant reduction in EE tissue area for all five angles of view from dorsal to ventral (*p* ≤ 0.019, non-parametric Kruskal–Wallis with Dunn’s post hoc test). Although mean EE tissue area was visibly reduced with both lower dsRNA concentrations (Figure 4i–p and Figure 6a), this reduction was only statistically significant for the 200 ng/µL treatment, in most but not all angles of view (*p* ≤ 0.047), in part due to statistical power when finely parsing our dataset sample sizes by angle of view. Despite this limitation, we were intrigued that in dorsal aspect we observed a consistent 40% reduction in mean EE tissue area compared to the wild type with all knockdown treatments (range: 36–44%), whereas reduction in ventral aspect ranged over two-fold (40–76%), in a dsRNA dose-dependent manner. Furthermore, the 200 ng/µL treatment reveals differing thresholds for degree of EE tissue reduction, with values comparable to the 100 ng/µL treatment (dorsal and dorsal-lateral), comparable to the 750 ng/µL treatment (lateral and ventral-lateral), or intermediate (ventral view).

To evaluate the non-uniform reduction in EE tissue after *Tc-zen1* RNAi, we also assessed EE domain size along both the A-P and D-V axes (Figure 6b). The wild-type serosa spans half the egg length dorsally and nearly the entire egg width in the anterior (Figure 6b,c: mean of 54% A-P and 94% D-V, compare with Figure 4a′,e′). In contrast, strong *Tc-zen1* RNAi embryos have a significantly reduced EE tissue area along both axes, but display a greater absolute reduction along the D-V axis (Figure 6b,c: mean of 31% A-P and 66% D-V; *p* < 0.0001 for both axes: Mann–Whitney U tests).

Altogether, this indicates a dose-dependent effect of *Tc-zen1* knockdown on the specification of EE area, with non-uniform positional changes in the EE–embryo tissue boundary.

### 3.6. Head Morphology Shows Conflicting Trends for Knockdown Phenotypic Response

**Figure 7:**
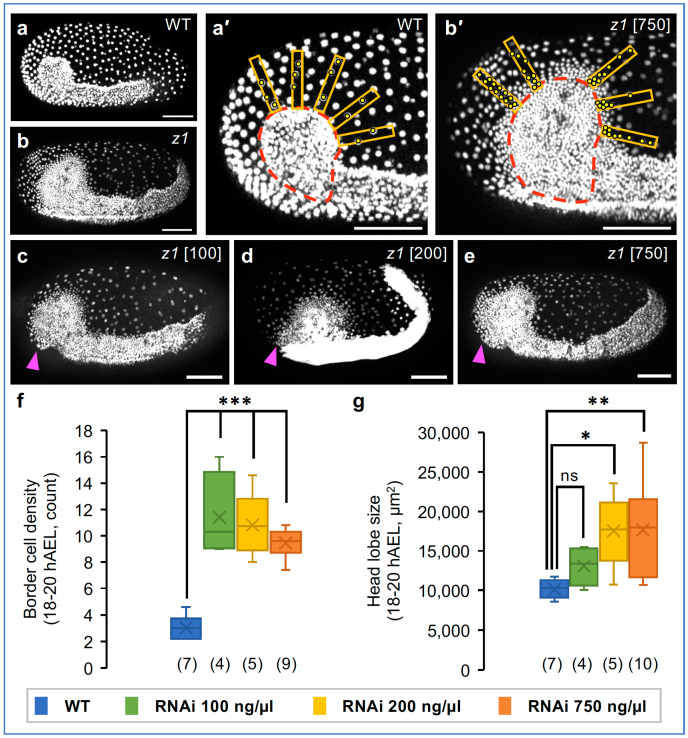
Evaluation of head size and peripheral cell packing. (**a**,**b**) Lateral aspect at 19 hAEL for WT (**a**) and strong *Tc-zen1* knockdown (**b**), depicting the extended germband embryo and widely spaced EE tissue. (**a′**,**b′**) Images annotated for the quantification of head size (dashed red outline) and cell packing (density) at the EE–embryonic border (orange rectangles with yellow nuclear counts: see Methods). (**c**–**e**) Stage-matched knockdown embryos (dsRNA concentrations as indicated), illustrating head size, head rim cell density, and the anterior-medial EE tissue flap (arrowheads). All scale bars are 100 µm. (**f**,**g**) Quantification of head rim cell density (**f**) and head area (**g**: single head lobe). Sample sizes are indicated parenthetically. All RNAi treatments were significantly different from WT but not from one another for border cell density. See Supplementary File S1 for raw values and test statistics ((**f**,**g**): one-way ANOVA and post hoc Tukey’s HSD).

If we have achieved weaker knockdown phenotypes with lower concentrations of *Tc-zen1* dsRNA, what are the implications for serosal vs. amniotic identity of the EE tissue? As the EE tissue structure resulting from complete loss of serosal identity is highly characteristic at germband stages (Figure 2 and Figure 3), we directly quantified these morphological hallmarks in our dilution series dataset (Figure 7a,b′).

With all three dsRNA concentrations, we find that knockdown embryos have (1) enlarged heads that (2) remain uncovered by EE tissue except in an anterior–medial flap and with (3) diffuse cell packing along the dorsal–lateral rim of the head lobes (Figure 7a–g).

For head rim cell packing, all three knockdown treatments were comparable to one another, with a significant, >3-fold increase in the border region cell density compared to wild-type embryos (Figure 7f). By this stage, in fact the wild-type embryo is fully enclosed by and physically separate from the outer serosa, while after *Tc-zen1* RNAi the head lobe ectoderm and EE tissue are contiguous within a cell sheet on the egg surface. Nonetheless, our quantification strategy addresses how compact, or diffuse, the embryonic tissue rim is, as well as the local cell density within the anterior EE tissue. Curiously, among the knockdown treatments, we see a counter-intuitive trend of a minor increase in head lobe rim delineation (a lower cell density due to a sharper tissue boundary transition) and with less inter-embryo variability with increasing concentrations of *Tc-zen1* dsRNA. Despite care in stage-matching embryos, these non-significant differences may reflect subtle differences in the developmental rate for head tissue condensation [1]. Alternatively, an intriguing possibility is that this may reflect delays due to greater ambiguity of tissue identity in the weaker knockdown conditions.

As with differentiated EE tissue area (Figure 6a), germband-stage head size reveals a graded response to knockdown, and again the 200 ng/µL treatment is more similar to the strong knockdown treatment (Figure 7g). The weakest knockdown treatment shows a modest 1.3-fold increase in head area compared to the wild type. Both the 200 ng/µL and 750 ng/µL treatments resulted in a more substantial, and significant, 1.7-fold increase in head size.

Taken together, the features of head rim cell packing and the absence of a lateral EE cover over the head lobes strongly distinguish all *Tc-zen1* knockdown treatments from the wild type. This suggests that the knockdown embryos feature a complete loss of serosal identity and that the EE territory comprises strictly amniotic tissue. However, the phenotypic outcome for head size supports a graded, dose-dependent knockdown response.

### 3.7. Tc-zen1 RNAi Influences the Rate of EE Polyploidization

Finally, we examined nuclear size dynamics as a fine-scale diagnostic feature of EE tissue identity, as nuclear size scales with ploidy under stage- and tissue-matched conditions (Figure 3l; [19,20,44]).

**Figure 8:**
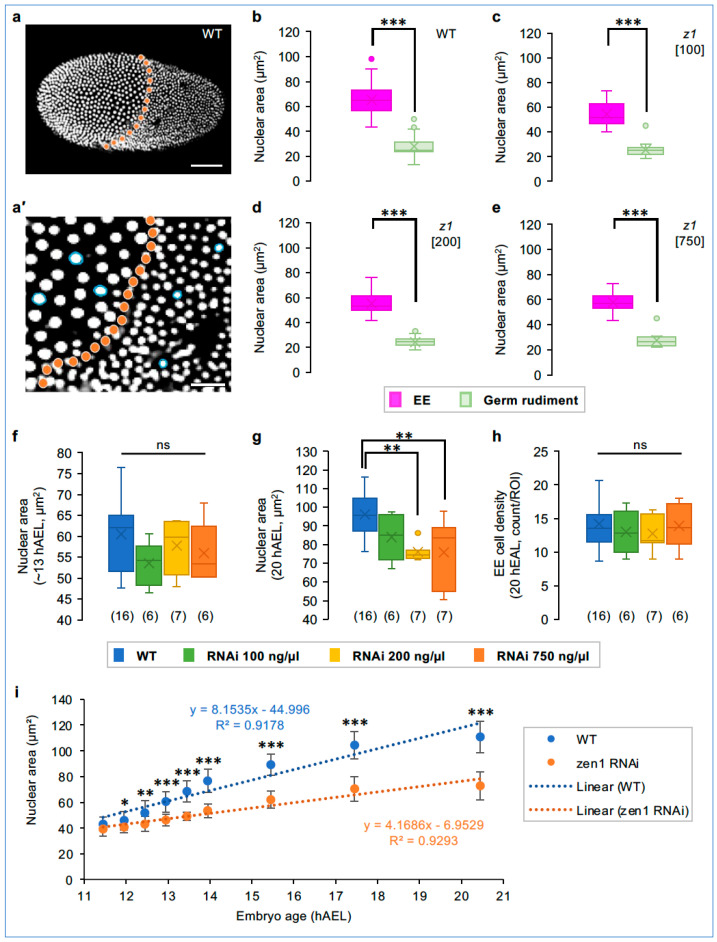
EE nuclear size dynamics as a proxy for tissue-specific polyploidization. (**a**,**a′**) At blastoderm differentiation, WT serosal nuclei (anterior, left) are larger than germ rudiment nuclei (posterior, right). The orange dotted line marks the tissue boundary; blue circles delineate selected nuclei (inset image; see Methods). Scale bars are 100 µm (**a**) and 25 µm (**a′**). (**b**–**e**) Nuclear area in the EE tissue and germ rudiment at the differentiated blastoderm stage (treatments indicated in the figure panels), with 3 nuclei measured per tissue region in 6–16 embryos per treatment (sample sizes as specified in panel (**f**)). (**f**,**g**) EE nuclear area at the differentiated blastoderm (13 hAEL) and extended germband (20 hAEL) stages, with 10 EE nuclei measured per embryo (sample sizes are indicated parenthetically; method schematic in Figure S1). (**h**) EE tissue cell density at 20 hAEL, as the mean nuclear count for three EE regions per embryo (each region 80 µm × 80 µm: method schematic in Figure S2). (**i**) EE nuclear size over time. For each treatment, 5 EE nuclei were tracked in 3 embryos (n = 15 nuclei/ treatment), plotted as mean ± standard deviation (see Figure S4 for individual plots). Equations for the linear trendlines and correlation coefficients (R^2^) are shown. See Supplementary File S1 for raw values and test statistics ((**b**–**e**): Mann–Whitney U; (**f**–**h**): one-way ANOVA; (**i**): *t*-test).

During wild-type blastoderm differentiation (about 13 hAEL), germ rudiment nuclei divide and become less than half the size of the non-dividing serosal nuclei (Figure 8a,a′,b). All knockdown treatments also show a significant, >2-fold nuclear size difference between the ATR and condensing embryonic tissue (Figure 8c–e). The EE nuclei in all knockdown treatments were about 8% smaller than the wild-type serosal nuclei, although this early difference was not significant (Figure 8f: wild-type mean of 61 µm^2^; knockdown EE means of 54–58 µm^2^; see Figure S1 for method). This confirms observations after strong knockdown (e.g., Figure 2g**′**), with the retention of larger nuclei in the ATR even with complete loss of serosal identity.

However, by the time of maximum germband extension (20 hAEL), we can detect significant differences in EE nuclear size (Figure 8g). Consistent with entry into the endocycle, EE nuclei increased in size in all cases, but to a lesser extent in the 200 ng/µL and 750 ng/µL treatments. In the wild-type and 100 ng/µL treatments, EE nuclei increased 1.6-fold, albeit with the RNAi sample still proportionately smaller. In contrast, in the two stronger knockdown treatments, EE nuclei increased in size by only 1.3-fold, attaining only 80% of wild-type serosal nuclear size (76 µm^2^, cf. 96 µm^2^). Despite this binary difference in EE nuclear size increase, the cell density within the EE tissue does not differ across treatments (Figure 8h and Figure S2).

Given the different EE nuclear sizes between the control and low-dsRNA compared to the medium- and high-dsRNA treatments by the stage of germband extension (Figure 8g and Figure S3), we analyzed the dynamics of nuclear size increase for each of these groups (Figure 8i and Figure S4). From 12 hAEL onward, wild-type serosal nuclei are significantly larger than the strong RNAi EE nuclei. Moreover, there is a 2.0-fold faster rate of nuclear size increase in the wild type throughout germband extension, heightened to 2.4-fold in a fast phase immediately after blastoderm differentiation (Supplementary File S1). In contrast, knockdown EE nuclei exhibit a modest, gradual increase, consistent with amnion-like nuclei that are ultimately smaller and display a weaker GFP fluorescent signal than the serosa (Figure 8i and Figure S3; [32]).

In sum, our live imaging quantification suggests that wild-type EE nuclei enter the endocycle at a faster cycle rate compared to after strong *Tc-zen1* knockdown. As EE ploidy levels are tissue-specific, this dynamic feature indicates that weak knockdown EE tissue is hybrid in character between bona fide serosa and amnion, exhibiting a serosa-like nuclear size increase (Figure 8g) in a tissue that is structurally amnion-like (Figure 7c,f and Figure 8h).

## 4. Discussion

The morphological appearances of cells and their genetic identities are normally coherent. For example, mammalian immune cells can be clearly distinguished by their nuclear morphology, degree of granulation, and relative size [74]. Furthermore, cell morphology can define functional state and predict subsequent fate, such as in pre-cancerous cells [75,76].

In this study, we use the insect EE epithelia as a tractable research system to explore (a) the limits of marker gene expression for assessing tissue identity after fate map changes in developing tissues and (b) how weaker RNAi phenotypes can reveal the effect of modulating a selector gene transcription factor on tissue identity and structure.

### 4.1. Uncoupled and Dose-Dependent Features of Tissue Identity

Genetic markers revealed the complete loss of serosal tissue identity after *Tc-zen1* RNAi (Figure 2 and Figure 3; [1,5]), but with two unexpected outcomes. First, the blastoderm ATR is genetically but not morphologically distinct from a serosal identity (Figure 2g,j). Second, altered gene expression in later development may reflect the *functional* transdifferentiation—not of overall tissue identity, but of morphogenetic character—between the serosa and amnion (Figure 2i; [13]).

Our live imaging of a knockdown dilution series directly tackles these ambiguities, revealing subtle properties of tissue patterning and specification dynamics. Similar to analysis of dsRNA concentration thresholds for embryonic phenotypes [77], our treatment conditions dissect nuances of EE tissue specification. The earliest features—the timing of blastoderm differentiation and EE nuclear size at differentiation (Figure 5a and Figure 8b–f)—are not predictive diagnostic features. However, both early EE and later embryonic tissue area support a graded phenotypic series after *Tc-zen1* knockdown (Figure 4, Figure 6a and Figure 7g). It is striking that these different measures of tissue area are concordant, considering the difference between the two-dimensional simplicity of the blastoderm and the later multilayered and folded tissue geometries. Similarly, nuclear size dynamics reveal a threshold between the 100 ng/µL and 200 ng/µL treatments for whether nuclei are serosa-like or amnion-like in the size and rate—but not onset—of polyploidization (Figure 8g,i). In addition, only the 750 ng/µL treatment showed a slowing in EE morphogenesis (Figure 5(b3)). This latter aspect likely reflects early secondary consequences of the largest shift to amniotic identity: amnion cells have a slower rate of cell shape change during embryonic gastrulation, presumably in part due to the loss of anterior *fog* expression after the loss of serosal identity [1,44,70]. Thus, a phenotypic series ranging from serosal to amniotic EE identity is supported by both cellular and tissue-scale properties, with specific tissue features sensitive to different levels of *Tc-zen1* knockdown.

Yet, mature tissue topology and structure are consistent with the complete loss of serosal identity in all knockdown treatments (Figure 7c–f). Upon reflection, this suggests a fundamental difference in nuclear size regulation between the amnion and serosa. Nuclei that are more amnion-like are smaller (Figure 3l, Figure 8g,i, Figures S3 and S4), but neither EE cell density near the head lobes nor throughout the tissue distinguishes between knockdown treatments (Figure 7f and Figure 8h). This is despite the fact that nuclear/cell scaling would be expected to favor a higher density for cells with smaller nuclei [22]. This suggests that a smaller nuclear size (lower ploidy) is a refractory, inherent feature of the amnion that does not respond to changes in cell packing and that can be uncoupled from other EE tissue features. Also, the folds of knockdown EE tissue (Figure 2i and Figure 7c–e) may provide flexible reservoirs that ensure that the dorsal yolk is fully covered by EE tissue, regardless of starting cell numbers.

Despite the existence of the knockdown ATR (Figure 2g,j), there is no equivalent subregionalization in the wild-type serosa (Figure S3; [5,13,51]). Furthermore, even with transitional EE tissue sizes after knockdown (Figure 4 and Figure 6), we still observed a clear EE–embryo tissue boundary. Also, nuclear size across the EE tissue exhibited no gradient that might imply mixed or progressive serosal/amniotic identities (Figure 4a**′**–h′, Figure 8b–e and Figure S3). A substantially weaker knockdown might produce transitional EE phenotypes. However, our observed phenotypes likely reflect binary EE/germ rudiment fate determination, integrating maternal genetic inputs.

### 4.2. Maternal Terminal Inputs Pattern Anterior Tissue Domains

As in *Drosophila*, the anterior pole of the *Tribolium* blastoderm is patterned by components of the terminal system. Maternal *Tc-torso-like* (*Tc-tsl*) activates zygotic *Tc-zen1*, *Tc-homeobrain* (*Tc-hbn*), and *Tc-decapentaplegic* (*Tc-dpp*) [2,55]. Additionally, an anterior maternal gradient of the conserved Wnt inhibitor *Tc-axin* suppresses posterior, embryonic Wnt signaling and thereby promotes anterior fates [2,4]. *Tc-hbn* and *Tc-zen1* then form a zygotic feedback loop, further suppressing posterior *Tc-caudal* (*Tc-cad*) and specifying the serosa [2]. The requirement of the terminal system to trigger this patterning cascade is further supported by the expansion of the serosal domain after the loss of the Torso pathway inhibitor *Tc-capicua* (*Tc-cic*) and its reduction after the loss of the Torso downstream target *Tc-maelstrom* (*Tc-mael*) [78,79]. Interestingly, while these factors affect the size of the serosal domain, their loss of function does not eliminate the serosa–germ rudiment tissue boundary, and none except *Tc-zen1* directly dictate serosal identity.

The majority of Torso pathway target genes remain to be identified [79]. One possible candidate is *Tc-hnt*, which retains ATR expression after *Tc-zen1* RNAi (Figure 2g). This was an unexpected result, as the wild-type EE expression of *Tc-hnt* is exclusively serosal [13,51]. However, comparison across species suggests that *hnt* expression dynamics involve *zen*-independent EE regulatory inputs for activation and spatial restriction. In *Drosophila*, the amnioserosal expression of *Dm-hnt* does not fully coincide with *Dm-zen* but only occurs in a nested subdomain [80]. In the jewel wasp *Nasonia vitripennis*, the earliest *Nv-hnt* expression is extensive and dynamic, before refining to the serosa in a domain that coincides with *Nv-zen* [81]. In the mosquito *Anopheles gambiae*, *Ag-zen* is strictly serosal, whereas early *Ag-hnt* expression spans the presumptive serosal and amniotic domains [40]. In the scuttle fly *Megaselia abdita*, *Ma-hnt* also spans the early serosal and amniotic domains [82]. Although EE *hnt* expression begins early in all species, functional studies mainly support a role in later morphogenesis, but not patterning, in *Tribolium* and fly species, in addition to conserved late roles in the peripheral nervous system [13,80,82].

Together, maternal factors initially pattern anterior terminal gene expression and morphology, and the extent of this is unmasked after knockdown of *Tc-zen1*. However, in light of the subsequent amnion EE identity after *Tc-zen1* knockdown, the anterior–terminal distinction represents a transient feature that is insufficient to dictate subsequent tissue identity.

### 4.3. A Selector Gene at the Nexus of Maternal Patterning and Zygotic EE Specification

The selector gene concept distinguishes transcription factors that are necessary and sufficient to confer specific cell and tissue identity. They can override the “ground plan” genetic landscape of target cells and act in parallel on cells with differing ground states, in order to pattern a complex tissue in a unified and functionally integrated manner [83,84]. For example, in murine neuronal stem cells, homeodomain selector genes act as global regulators across a complex regulatory landscape in their target cell types [85].

Classic examples of selector genes are the Hox genes that confer segment-specific identity along the anterior–posterior body axis of the embryo [86,87,88]. The diverged insect *Hox3* orthologue, *zen*, or *zen1*, no longer functions in the embryo, and yet these genes retain conserved Hox-like sequence features and are recognized as having selector gene function in the extraembryonic domain [37,48,89,90]. Hence, despite the complexity of maternal anterior patterning through multiple signaling pathways and other transcription factors, zygotic Zen alone is known to determine serosal tissue identity. In the fly *Megaselia*, ectopic *Ma-zen* promotes serosal development, in part through repression of amniotic marker genes that respond to different thresholds of *Ma-zen*; and in *Drosophila*, prolonged overexpression of *Dm-zen* leads to enlarged nuclei in the amnioserosa [82].

Similarly, in *Tribolium*, we show here that while hallmark serosal cell morphology may originate independently of *Tc-zen1* activity in the ATR, this alone is insufficient to produce bona fide serosal identity (Figure 2 and Figure 3), and neither does this ground state alter the mature tissue structure within the wild-type serosa (Figure S3). Thus, full serosal identity overrides the initial distinction of the ATR subregion. On the other hand, although the hybrid cell character in our weakest knockdown treatment exhibited a serosa-like nuclear size increase (Figure 8 and Figure S3), ultimately the tissue has amnion-like properties akin to the strong knockdown condition, including for integrated measures of nuclear morphology (Figure 7 and Figure S3c). 

In light of serosal tissue biology and function [5,25,59], we have uncovered suites of dynamic developmental traits with different sensitivities to Tc-Zen1 activity. Whereas features such as cuticle secretion require high levels of Tc-Zen1, the initial nuclear size increase—but not the full extent of polyploidization—responds to very low doses. As in studies on fly limb and sensory organ patterning [91,92], we thus find that the importance of the selector gene as a master regulator is modulated by interplay with its wider genetic network, to ultimately pattern the full spectrum of tissue identity.

## Data Availability

The data presented in this study are available in the article and the Supplementary Materials.

## Supplementary Materials

The following supporting information can be downloaded at: https://www.mdpi.com/article/10.3390/cells13141211/s1. Within the supplementary PDF: Figure S1, Measuring EE nuclear area during blastoderm differentiation; Figure S2, Measuring cell density of the EE tissue at 20 hAEL; Figure S3, EE nuclear features mapped along the A-P axis at 19 hAEL; Figure S4, Nuclear area over time for WT and strong *Tc-zen1* knockdown embryos; Table S1, Primers used in this study. Additional Supplementary Files: Supplementary File S1; Raw data values and statistical tests for Figure 5, Figure 6, Figure 7 and Figure 8 [xlsx file]; Movie S1, Time-lapse movie of wild-type dorsal closure in a heterozygous cross for serosal and cardioblast GFP, as in Figure 3h [avi file]; Movie S2, Time-lapse movie of dorsal closure after *Tc-zen1* parental RNAi, in a heterozygous cross for serosal and cardioblast GFP, as in Figure 3j [avi file]; Movies S3–S7, Representative time-lapse movies of early embryogenesis in the ubiquitous nuclear-GFP background for the wild type (Movie S3) and each of the three *Tc-zen1* dsRNA concentrations (Movie S4: 750 ng/µL; Movie S5: 200 ng/µL; Movies S6–S7: 100 ng/µL), from blastoderm differentiation through germband extension, as in Figure 4 and Figure 7 [avi files] (additional movie legend information provided in the Supplementary PDF).
