# Supplementary material for: Tissue-Level Integration Overrides Gradations of Differentiating Cell Identity in Beetle Extraembryonic Tissue
Source: Cells. 2024 Jul 18;13(14):1211. doi: 10.3390/cells13141211 (PMC11274815; doi:10.3390/cells13141211)
Supplement: Supplementary file 1 [file cells-13-01211-s001.zip › Supplementary-Information-z1-series.pdf]

Tissue-level integration overrides gradations of differentiating cell identity in beetle extraembryonic tissue

Supporting Information

In this PDF:

Fig. S1. Measuring EE nuclear area during blastoderm differentiation.

Fig. S2. Measuring cell density of the EE tissue at 20 hAEL.

Fig. S3. EE nuclear features mapped along the A-P axis at 19 hAEL.

Fig. S4. Nuclear area over time for WT and strong Tc-zen1 knockdown embryos.

Table S1. Primers used in this study.

Additional supplementary files:

Supplementary File S1. Raw data values and statistical tests from Figures 5-8.

Movie S1. Time-lapse movie of wild type dorsal closure in a heterozygous cross for serosal and cardioblast GFP, as in Fig. 3h.
Posterior embryonic fluorescence includes the proctodeum (also visible in Fig. 3k), which flexes ventrally out of the field of view as dorsal closure progresses.

Movie S2. Time-lapse movie of dorsal closure after *Tc-zen1* parental RNAi, in a heterozygous cross for serosal and cardioblast GFP, as in Fig. 3j.
There is minor fluorescent signal of yolk components, but a complete absence of serosal GFP. Without the contractile force of the serosa, dorsal closure is slower and less efficient than in wild type [1].

Movies S3-S7. Representative time-lapse movies of early embryogenesis in the ubiquitous nuclear-GFP background for wild type (Movie 3) and each of the three *Tc-zen1* dsRNA concentrations (Movie 4: 750 ng/µl; Movie 5: 200 ng/µl; Movies 6-7: 100 ng/µl), from blastoderm differentiation through germband extension, as in Figs. 4 and 7.
Shown in lateral aspect (Movies 3-6) or dorsal-lateral aspect (Movie 7). Note that fixed brightness settings on these export files leads to some overexposure of embryonic tissue in later stages, whereas in the original high resolution files brightness is within the acquisition dynamic range for visualizing the EE nuclei at all stages and for embryonic tissue at early stages (*e.g.*, Fig. S4b). The loss of peripheral signal in the last few hours of Movie 6 was due to a drop in focal plane of acquisition, not a loss of tissue in the embryo. Time stamps indicate minimum age from a 1-hour range, with recordings made at 26-28 ºC.

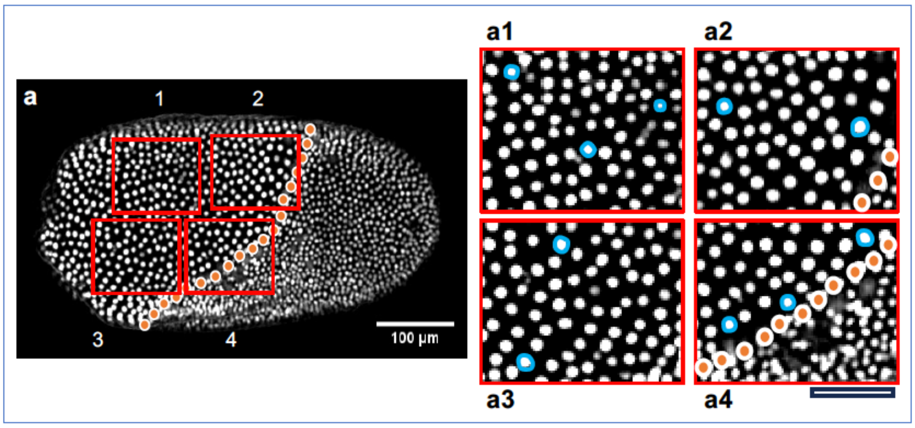

Fig. S1. Measuring EE nuclear area during blastoderm differentiation.
For each embryo, four rectangular regions (125 µm x 100 µm) were placed on the EE tissue territory and a total of 10 nuclei (2-3 nuclei per region) were chosen at random for quantification (inset images a1-a4), illustrated here with a wild type control embryo**.** At the extended germband stage (20 hAEL), nuclei covering the yolk were selected. Scale bar for all inset images is 50 µm. Supports Fig. 8f-g.

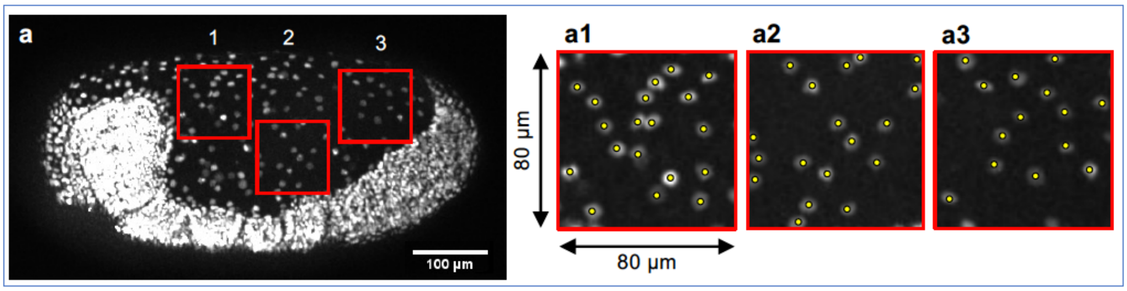

Fig. S2. Measuring cell density of the EE tissue at 20 hAEL.
For each embryo, three square regions (80 µm x 80 µm) were placed on the EE tissue territory and individual nuclei were counted, for all nuclei with >50% of nuclear area within the bounding boxes (inset images a1-a3), illustrated here with an embryo from the strong *Tc-zen1* RNAi treatment (750 ng/µl). The mean nuclear count from the three regions was recorded as the EE cell density value per embryo. Supports Fig. 8h.

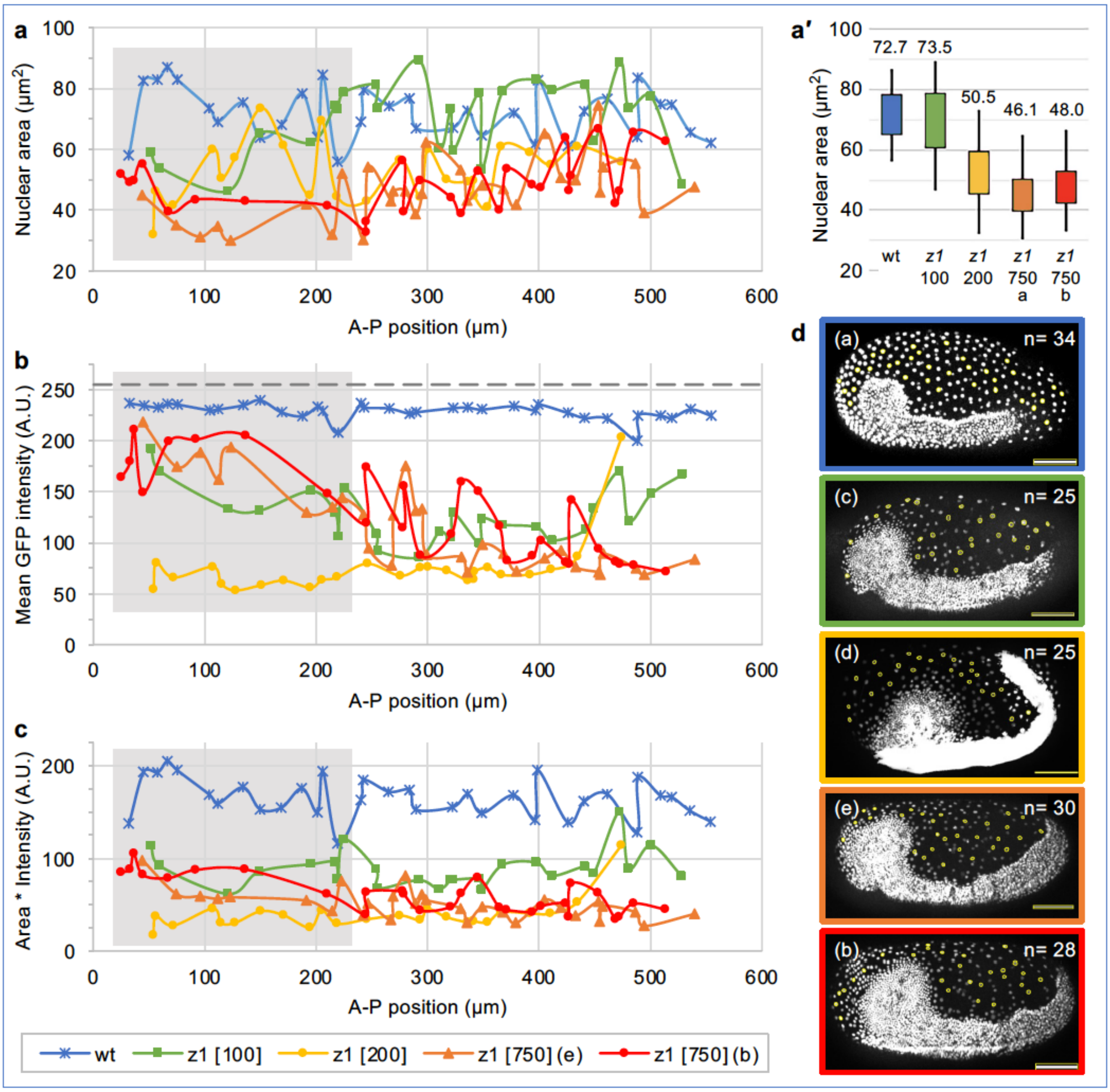

Fig. S3. EE nuclear features mapped along the A-P axis at 19 hAEL.
For each embryo, ≥25 EE nuclei were measured for size (**a**: area, µm^2), mean GFP intensity (**b**: grayscale range of 0-255, with saturation at 255 indicated by the dashed black line), and for area weighted for intensity (**c**), based on the representative embryos for each treatment that are featured in main text Fig. 7 (**d**: corresponding main text image panel and nuclear sample sizes are indicated, with yellow circles for the measured nuclei). Additionally, for nuclear area the box plot (**a′**) depicts the interquartile range and minimum and maximum individual values, with the median reported within the chart. In (a-c), the grey shaded region indicates the position of the embryonic head lobes. Note that brightness settings are optimized for consistent, clear signal in the border region at the rim of the head lobes, leading to ostensible oversaturation in thick, multinuclear germband embryonic tissue, particularly as knockdown EE nuclei generally have weak fluorescence.

Knockdown EE nuclei near embryonic tissue (nuclei measured in the most anterior and posterior regions) tend to be brighter (b), but not to differ in size compared to EE nuclei elsewhere in the tissue (a). Overall, nuclear area alone distinguishes between knockdown treatments, with the weakest knockdown comparable to wild type serosal nuclear size (a, a′), as in Fig. 8g; GFP intensity sharply distinguishes wild type serosal nuclei by brightness and homogeneity compared to all knockdown EE nuclei (b); while area weighted by GFP intensity most consistently distinguishes phenotypic categories for wild type, the weakest knockdown (100 ng/µl dsRNA), and stronger knockdown (200 or 750 ng/µl dsRNA) treatments. Supports Fig. 8f-g.

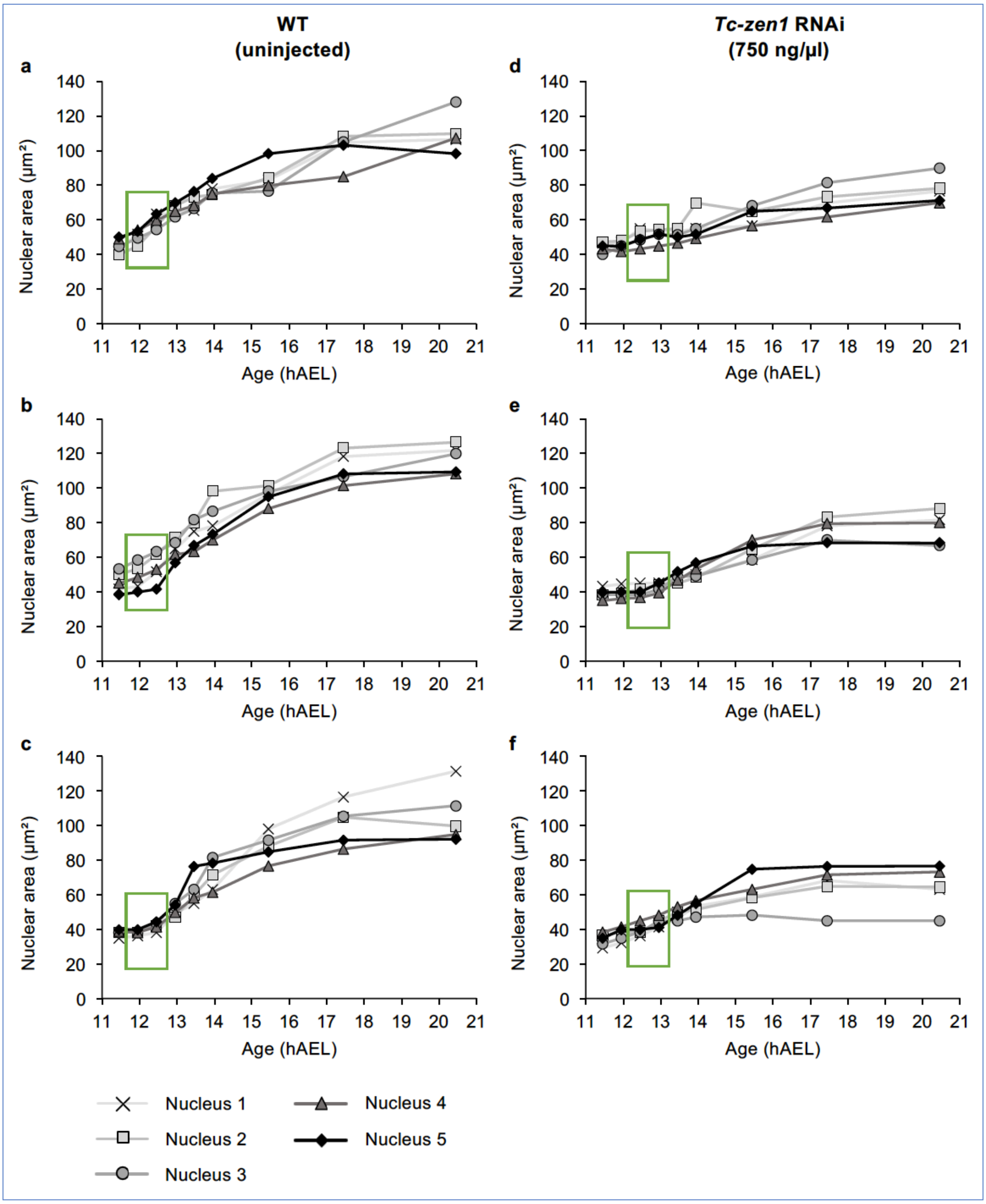

Fig. S4. Nuclear area over time for WT and strong *Tc-zen1* knockdown embryos. Individual values for changes over time in nuclear area, for five tracked EE nuclei per embryo, in each of three embryos for the WT **(a-c)** and *Tc-zen1* RNAi 750 ng/µl **(d-e)** treatment conditions. Nuclei were tracked from 11.45 hAEL (prior to differentiation of the blastoderm) to 20.45 hAEL (stage of maximum germband extension), at nine selected time points. The green boxes demarcate the exact timing of blastoderm differentiation in each embryo. These data support Fig. 8i, where the average of the 15 nuclei per treatment is plotted with the standard deviation.

Table S1. Primers used in the study. All primers also included either the 5' adapter sequence 5'-GGCCGCGG-3' (forward primers) or the 3' adapter sequence 5'-CCCGGGGC-3' (reverse primers) for subsequent amplification with T7 promoter universal primers (adapters not shown in table). The T7 adapter primers are: 5'-universal primer 5'-GAGAATTCTAATACGACTCACTATAGGGCCGCGG-3', and 3'-universal primer 5'-AGGGATCCTAATACGACTCACTATAGGGCCCGGGGC-3'.

Application	Gene and fragment ID	Primer direction	Sequence (5' to 3')	Amplicon length (bp)
**RNAi**				
	Tc-zen1 (TC000921)	forward	TTTGAAAACCAAGCCGTTCT	203
	(short fragment, eRNAi for live imaging)	reverse	CGTTGGGGTTGAGTTTCTTG	
	Tc-zen1 (TC000921)	forward	TCCCAATTTGAAAACCAAGC	688
	(long fragment, pRNAi for *in situ* hybridization)	reverse	CGTTCCACCCTTCCTGATAA	
	Tc-dup (TC003416)	forward	GGCTTCAAACGAATCCAAAA	373
		reverse	GGTCTTCAACTGTGGCGATT	
***in situ* hybridization**				
	Tc-hnt (TC009560)	forward	TGACTTGACCAAGACGCAAG	627
		reverse	GCTTTCTTGACCTCCTCACG	
	Tc-pnr (TC010407)	forward	ATGCTTGTGGGCTTTACCAC	757
		reverse	GCAGTAACGTGGTGTTGGTG	

REFERENCE

1. Panfilio, K.A., Oberhofer, G., and Roth, S. (2013). High plasticity in epithelial morphogenesis during insect dorsal closure. Biol. Open *2*, 1108-1118.
